# Impact of the glutathione synthesis pathway on sulfasalazine-treated endometrial cancer

**DOI:** 10.18632/oncotarget.28185

**Published:** 2022-01-26

**Authors:** Kanako Sendo, Manabu Seino, Tsuyoshi Ohta, Satoru Nagase

**Affiliations:** ^1^Department of Obstetrics and Gynecology, Yamagata University Faculty of Medicine, Yamagata, Japan

**Keywords:** endometrial cancer, glutathione, sulfasalazine, xCT, cystathionine gamma-lyase

## Abstract

Glutathione is an antioxidant that has an important role in chemotherapeutic drug resistance in cancer. Cysteine is synthesized from cystine and is transported into the cell via the xCT antiporter. Another pathway for synthesizing cysteine involves intracellular methionine. We determined whether targeting the xCT represents a promising strategy for the treatment of endometrial cancer and identified factors that predict efficacy of this treatment strategy. In uterine serous carcinoma (USC) cell lines, the combination of cisplatin and the xCT inhibitor, sulfasalazine, significantly inhibited cell growth compared with single-agent cisplatin or sulfasalazine. Sulfasalazine treatment significantly decreased intracellular glutathione levels and induced apoptosis when combined with cisplatin in USC cell lines. On the one hand, the effectiveness of combined cisplatin and sulfasalazine was not evident in endometrioid carcinoma. USC cell lines exhibited increased expression of xCT and decreased expression of cystathionine gamma lyase (CGL), which is an enzyme involved in the synthesis of cysteine from methionine. On the other hand, endometrioid carcinoma cell lines exhibited increased CGL expression or decreased xCT expression. These findings suggest that using a glutathione synthesis pathway-based approach for selecting subjects for sulfasalazine treatment may be an effective strategy for circumventing glutathione-related chemotherapeutic drug resistance in endometrial carcinoma.

## INTRODUCTION

Endometrial cancer is a common gynecologic malignancy. Most endometrial cancers are classified as early stage and low grade [e.g., endometrioid carcinoma (EmC) grade 1, 2] and are cured with surgery alone. Endometrial cancer is generally classified into two categories, type I and type II, based on histopathologic features. Type I endometrial cancer, consisting of grade 1 (G1) and grade 2 (G2) endometrioid carcinoma, is estrogen-dependent, accounts for 80% to 90% of all endometrial cancers, and has a 5-year survival rate greater than 85% [1]. It usually occurs in pre- and peri-menopausal women and is associated with obesity, hyperlipidemia, and hyperestrogenism. In contrast, type II endometrial cancer, including serous and clear cell carcinoma, is estrogen-independent and is not associated with hormonal factors. Patients with type II endometrial cancer have a poor prognosis, a 5-year survival rate of 55%, and a recurrence rate of 44% [1, 2]. Among the type II endometrial cancers, uterine serous carcinoma (USC) is the most common subtype. Although USC only accounts for 10% of all endometrial cancer cases, it is responsible for 39% of the deaths [3]. The 5-year survival of USC is also poor (27–55%) compared with that of low grade endometrial cancer subtypes [3, 4]. USC demonstrates a high rate of recurrence following initial treatment and is estimated to be 21–35.8% [5, 6] and 82.7% of the cases present at advanced stages, III-IV [7]. This disproportion indicates the need for improvements in USC diagnosis and treatment. Recent studies have shown that chemotherapy likely has a benefit in advanced-stage and recurrent endometrial cancers [8]. Although cisplatin (CDDP) is a cornerstone drug used for endometrial cancer treatment [8], several drug resistance mechanisms have been reported, including enhanced drug efflux by multidrug resistance protein 2 [9], DNA repair associated with tyrosine kinase with immunoglobulin-like and EGF-like domains 1 (TIE1) [10], and Reactive oxygen species (ROS) scavenging by glutathione (GSH) [11]. Thus, novel therapeutic targets for chemotherapy-resistant USC are needed.

Metabolomic analysis is a recently developed technique for evaluating biological specimens based on various metabolic pathways. Metabolites produced from metabolic pathways are involved in a variety of tumorigenic processes. A metabolomic approach revealed part of the mechanism for platinum resistance in ovarian cancer [12]. Previously, we demonstrated that GSH concentration in paclitaxel-resistant USC cells is higher compared with that in parental cells. The results indicated that increased GSH may be related to the acquired drug resistance phenotype and may represent a therapeutic target [13]. GSH, a tripeptide composed of glutamate (Glu), cysteine (Cys), and glycine (Gly), is one of the main antioxidants in cancer cells. ROS produced by chemotherapeutic drugs can induce cell death [14]; therefore, high levels of GSH may contribute to cancer cell survival and resistance to chemotherapy. GSH synthesis is regulated by Glu-Cys ligase catalytic subunit (GCLC) activity, Cys availability, and GSH feedback inhibition [15]. Cys is imported into cancer cells through the Glu-Cystine transporter, xCT, or derived from methionine (Met) by the trans-sulfuration pathway. The expression of enzymes related to the trans-sulfuration pathway varies in tumor specimens and cell lines [16–18]. In contrast, xCT expression is detected in most tumor tissues and cell lines [16]. The Glu-cystine transporter, xCT, is important for GSH synthesis; therefore, xCT is a potential target for anticancer therapy.

Sulfasalazine (SAS) has been widely reported as an inhibitor of xCT. There has been a long-standing clinical history using SAS for the treatment of rheumatoid arthritis and inflammatory bowel disease. Several reports have demonstrated efficacy of SAS in enhancing the effectiveness of chemotherapeutic drugs against various cancers, such as colorectal cancer, bladder cancer, and melanoma [19–21]. However, it remains unclear whether xCT inhibition can circumvent GSH-mediated resistance to anticancer therapy in endometrial cancer. In this study, we determined whether SAS enhances the efficacy of CDDP and identified factors that influence the efficacy of SAS in endometrial cancer.

## RESULTS

### Effect of SAS on intracellular GSH and the efficacy of CDDP in USC and EmC cell lines

**Figure 1:**
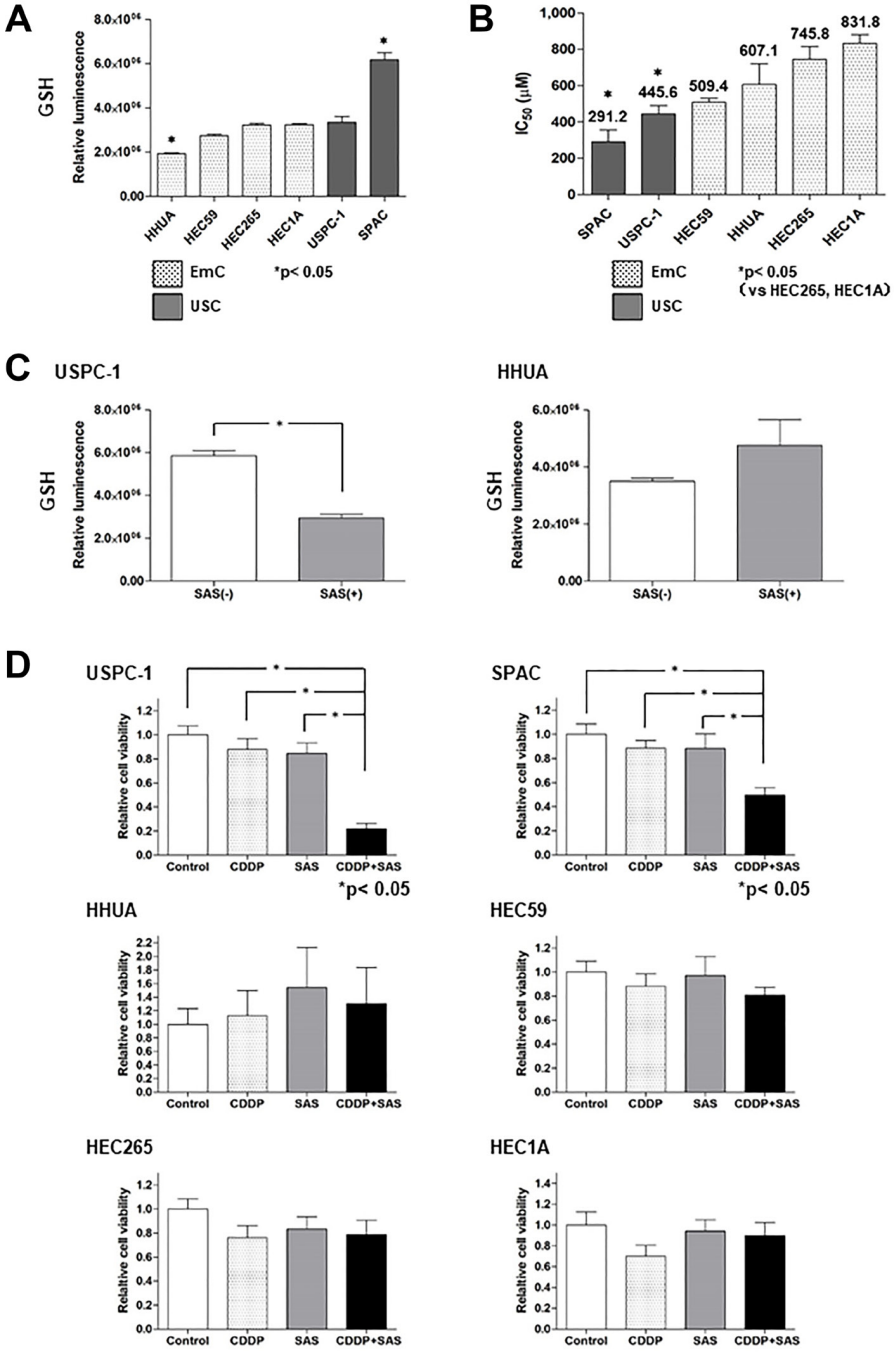
Sulfasalazine (SAS) enhances the efficacy of cisplatin (CDDP) in uterine serous carcinoma (USC) cells resulting from the depletion of intracellular glutathione (GSH). (**A**) Intracellular GSH concentration of endometrial cancer cell lines. Values shown represent means + SD. (**B**) The IC_50_ values of SAS for the cell lines were calculated as described in the Materials and Methods. Values shown represent means + SD. (**C**) Intracellular GSH concentration in USPC-1 and HHUA cells was measured before and after a 48-h treatment with 400 μM SAS. Values shown represent means + SD. ^*^ *P* < 0.05. (**D**) USPC-1 cells were treated with 3 μM CDDP and 400 μM SAS for 48 h. Subsequently, cell viability was assessed using the MTS assay. SPAC, HHUA, HEC59, HEC265, and HEC1A cells were treated with 2 μM CDDP and 100 μM SAS, 0.5 μM CDDP and 300 μM SAS, 3 μM CDDP and 200 μM SAS, 5 μM CDDP and 300 μM SAS and 10 μM CDDP, and 300 μM SAS, respectively. Values shown represent means + SD. ^*^ *P* < 0.05.

We first evaluated intracellular GSH levels in endometrial cancer. GSH levels in USC cells (USPC-1, SPAC) tended to be higher compared with that in EmC cells (HHUA, HEC59, HEC265, HEC1A) (Figure 1A). Next, we examined the effect of SAS on cell viability in USC and EmC cells. Further titrations revealed half-maximal inhibitory concentration (IC_50_) values of 291.2 μM and 445.6 μM in SPAC and USPC-1, respectively, following treatment with SAS. IC_50_ values of 509.4, 607.1, 745.8, and 831.8 μM were observed for HEC59, HHUA, HEC265, and HEC1A, respectively. SAS inhibited cell proliferation more effectively in USC cells compared with that in EmC cells (Figure 1B). To confirm that SAS inhibits xCT-mediated cystine transport, we measured GSH levels in USPC-1 and HHUA cells before and after treatment with SAS. Treatment with 400 μM SAS for 48 h resulted in a marked depletion of intracellular GSH levels in USPC-1 cell lines (Figure 1C, left), whereas a 48-h treatment with 400 μM SAS increased GSH levels in HHUA cells (Figure 1C, right). Finally, we examined whether SAS affected CDDP cytotoxicity by treating cells with CDDP alone or in combination with SAS for 48 h. We conducted these experiments using the respective IC_50_ values of SAS and CDDP for each cell line. CDDP plus SAS significantly inhibited cell proliferation compared with CDDP or SAS alone in USC cell lines. In contrast, SAS showed no effect on CDDP cytotoxicity in EmC cell lines (Figure 1D). Collectively, these results suggest that SAS-induced growth inhibition of USC cells, but not EmC cells, and enhanced the efficacy of CDDP in USC cells because of a depletion of intracellular GSH levels.

### ROS accumulation and apoptosis in USC cell lines treated with CDDP and/or SAS

**Figure 2:**
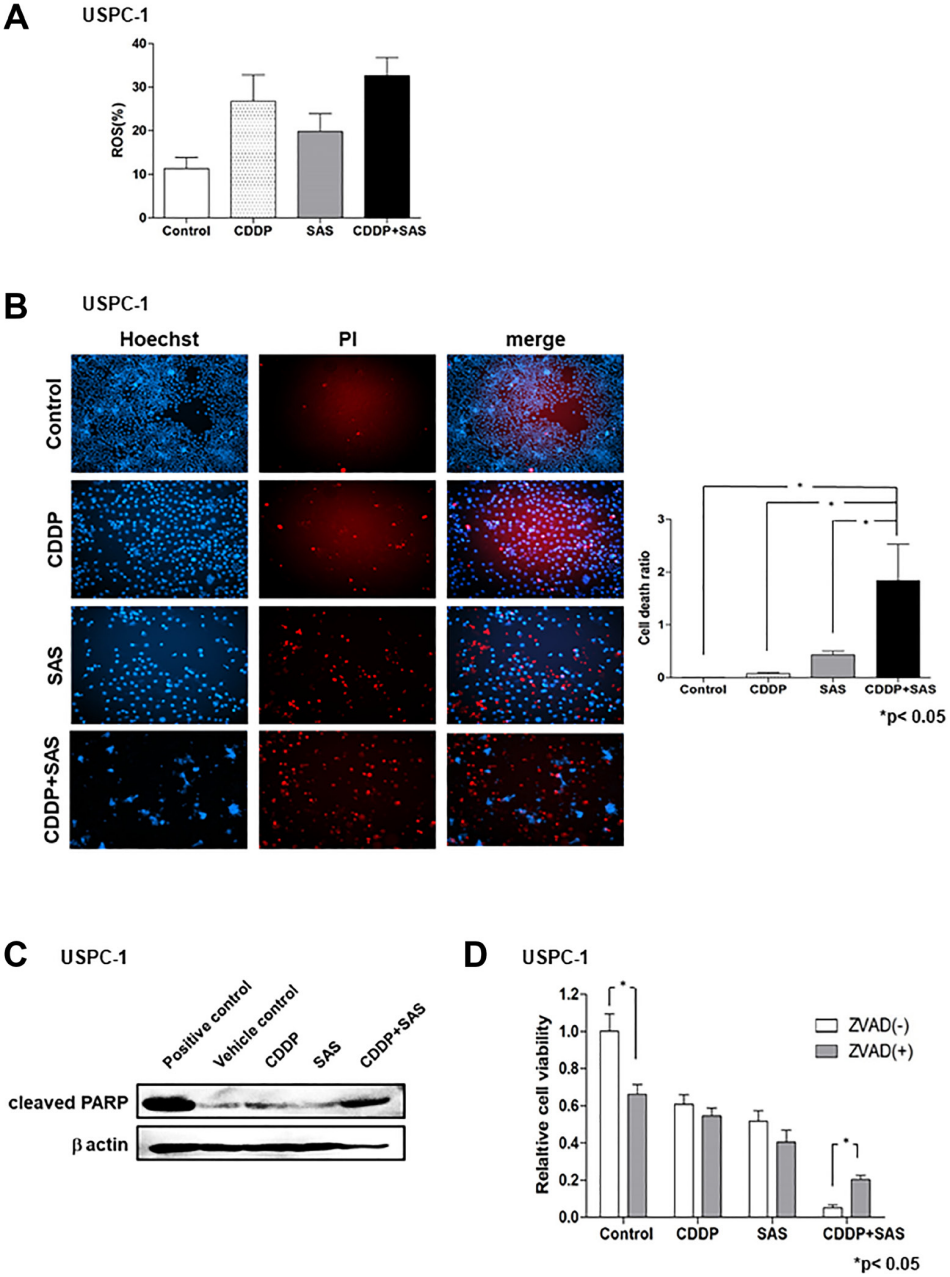
Sulfasalazine (SAS) plus cisplatin (CDDP) accumulate reactive oxygen species (ROS) and induces apoptosis. (**A**) USPC-1 cells were cultured for 48 h in the presence of 3 μM CDDP and/or 400 μM SAS. Flow cytometry was used to measure intracellular ROS levels after staining with DCFH-DA. (**B**) USPC-1 cells were treated with 3 μM CDDP and/or 400 μM SAS for 48 h and then used for cell death assays performed using PI as a vital dye and Hoechst nuclear staining. Values shown represent means + SD. ^*^ *P* < 0.05. (**C**) USPC-1 cells were treated with 3 μM CDDP and/or 400 μM SAS for 48 h, and cell lysates were immunoblotted with an anti-cleaved PARP antibody. (**D**) USPC-1 cells were cultured for 2 h in the absence or presence of 100 μM Z-VAD-FMK and then treated for 48 h with 3 μM CDDP and/or 400 μM SAS. Subsequently, cell viability was assessed using the MTS assay. Values shown represent means + SD. ^*^ *P* < 0.05.

We next measured intracellular ROS levels in USC cells treated with CDDP and/or SAS by FACS analysis using 2′,7′-dichlorofluorescin diacetate (DCFH-DA). CDDP plus SAS increased intracellular ROS levels compared with treatment with CDDP or SAS alone (Figure 2A). ROS can cause the collapse of antioxidant systems and thereby lead to cell death [22]. To examine whether SAS induces USC cell death, we performed cell death assays using the fluorescent vital dye, propidium iodide (PI). PI staining (Figure 2B, right) revealed that SAS and/or CDDP induced significant cell death in USPC-1 cells. CDDP plus SAS significantly increased cell death compared with treatment using CDDP or SAS alone (Figure 2B, left). SAS has been widely reported to induce cell death and tumor growth inhibition through apoptosis in colorectal cancer and head and neck squamous cell carcinoma [23, 24]. Therefore, we measured the expression of an apoptotic marker, PARP, by immunoblotting with an anti-cleaved PARP antibody. Immunoblot analysis revealed that CDDP plus SAS increased PARP cleavage compared with that in cells treated with CDDP or SAS alone (Figure 2C). To further confirm that SAS-induced cell death occurred through apoptosis, we treated cells with CDDP and/or SAS plus the apoptosis inhibitor, Z-VAD-FMK, and found that the inhibitor reversed CDDP plus SAS-induced cell death (Figure 2D). These results indicate that CDDP plus SAS enhances CDDP-mediated ROS accumulation and apoptosis in USC cells.

### Effect of BSO on intracellular GSH and ROS, and the efficacy of CDDP in USC and EmC cell lines

**Figure 3:**
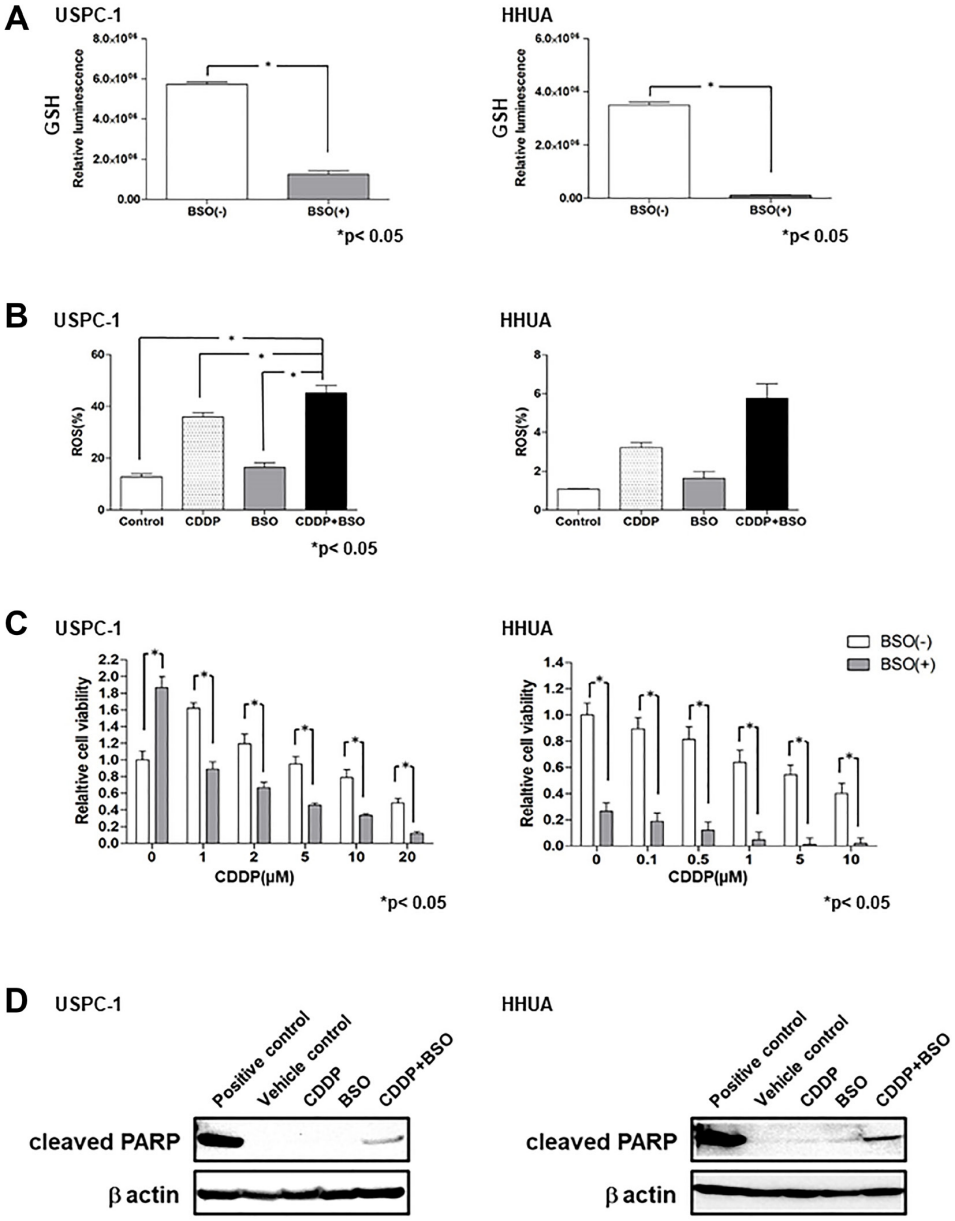
Buthionine sulfoximine (BSO) decreases intracellular glutathione (GSH) levels and inhibits cell proliferation with cisplatin (CDDP) in endometrial carcinoma cell lines. (**A**) Intracellular GSH concentration of USPC-1 and HHUA cells treated with 100 μM BSO for 48 h. Values shown represent means + SD. ^*^ *P* < 0.05. (**B**) USPC-1 cells treated with 3 μM CDDP and/or 100 μM BSO and HHUA cells treated with 0.5 μM CDDP and/or 100 μM BSO for 48 h. Intracellular ROS levels were measured using flow cytometry after staining with DCFH-DA. Values shown represent means + SD. ^*^ *P* < 0.05. (**C**) USPC-1 and HHUA cells were treated with 100 μM BSO and the indicated concentrations of CDDP for 48 h. Subsequently, cell viability was assessed using the MTS assay. Values shown represent means + SD. ^*^ *P* < 0.05. (**D**) USPC-1 cells were treated with 3 μM CDDP and/or 100 μM BSO for 48 h, and cell lysates were immunoblotted with an anti-cleaved PARP antibody. HHUA cells were treated with 0.5 μM CDDP and/or 100 μM BSO for 48 h, and cell lysates were immunoblotted.

Uptake of cystine by xCT provides most of the cellular Cys, which is eventually converted to GSH, but a significant percentage is derived from Met through the trans-sulfuration pathway. GCLC converts Cys to γ-glutamylcysteine, which is a precursor substrate for GSH. We examined the effect of the GCLC inhibitor, buthionine sulfoximine (BSO), on intracellular GSH levels in USC and EmC cell lines. Although the xCT inhibitor, SAS, exhibited no depletion of GSH in HHUA cells, treatment with 100 μM BSO for 48 h resulted in a marked depletion of intracellular GSH levels in USPC-1 and HHUA cells (Figure 3A). These results suggest that intracellular GSH levels may be reliant upon the trans-sulfuration pathway in EmC cells. We next evaluated intracellular ROS levels in USC and EmC cells treated with CDDP and/or BSO by FACS analysis. CDDP plus BSO increased intracellular ROS levels compared with treatment with CDDP or BSO alone in both cell lines (Figure 3B). We tested whether BSO affected CDDP-mediated cytotoxicity by treating cells with CDDP alone or in combination with BSO for 48 h. CDDP plus BSO significantly reduced cell viability compared with CDDP alone at each concentration in both cell lines (Figure 3C). Next, we examined whether co-treatment with CDDP and BSO could enhance apoptosis compared with single-agent treatment of CDDP or BSO. Immunoblot analysis revealed that CDDP plus BSO increased PARP cleavage compared with that in cells treated with CDDP or BSO alone (Figure 3D). These results suggest that BSO enhanced CDDP-mediated cytotoxicity in not only USC cell lines, but also EmC cell lines through GSH depletion and ROS accumulation.

### The underlying mechanism between the GSH synthesis pathway and the effect of SAS

**Figure 4:**
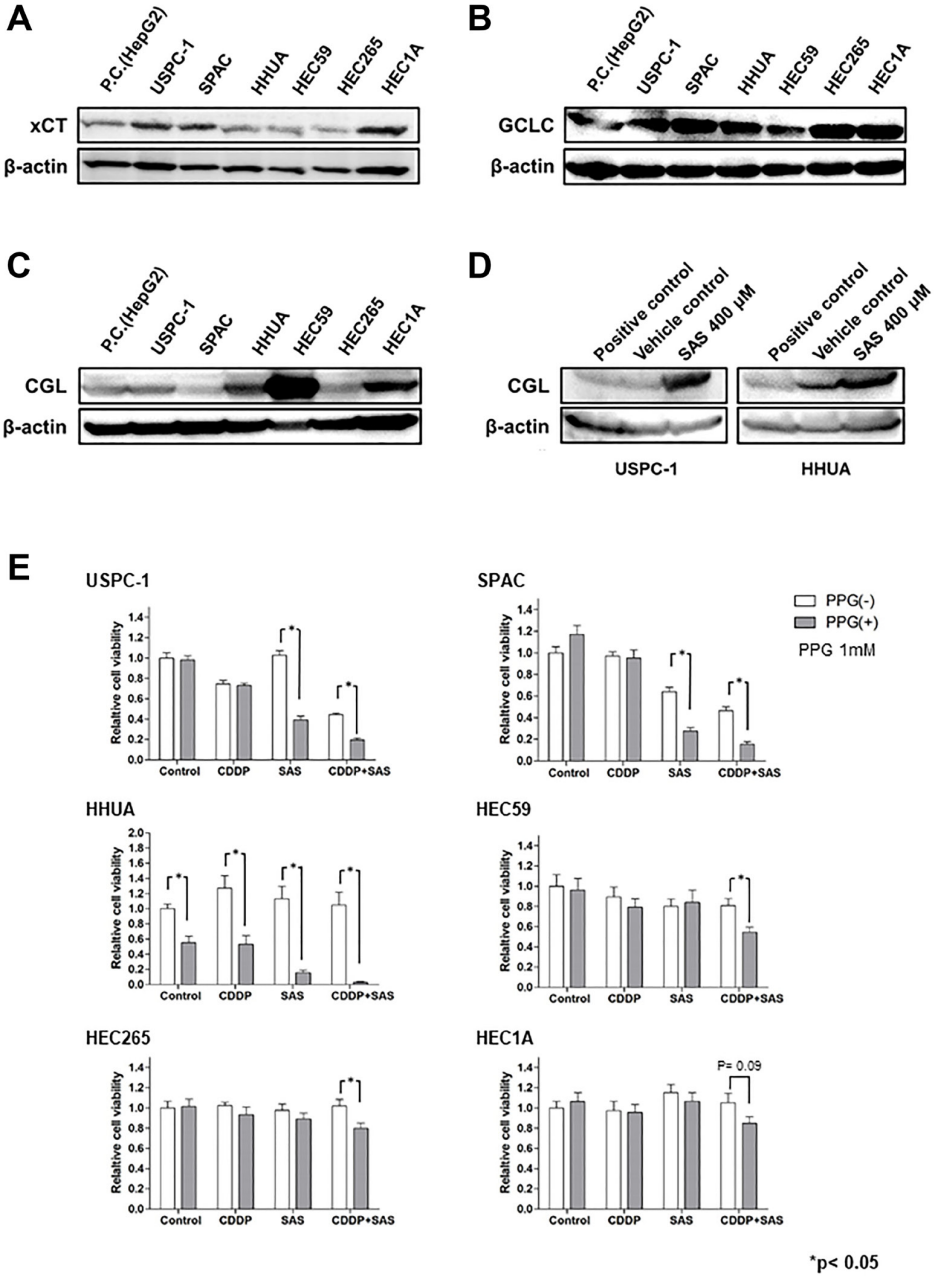
The glutathione (GSH) synthesis pathway is associated with the effect of Sulfasalazine (SAS). (**A**–**C**), Expression of xCT, Glu-Cys ligase catalytic subunit (GCLC), and cystathionine gamma lyase (CGL) were measured in USPC-1, SPAC, HHUA, HEC59, HEC265, and HEC 1A cells. Cell lysates were analyzed by western blot analysis using an anti-xCT, anti-GCLC, and anti-CGL antibody. B-actin was used as an internal control. (**D**) USPC-1 and HHUA cells were treated with 400 μM SAS for 48 h, and cell lysates were immunoblotted with an anti-CGL antibody. (**E**) USPC-1 cells were treated with 3 μM CDDP and 400 μM SAS for 48 h with or without 1 mM PPG. Subsequently, cell viability was assessed using the MTS assay. HEC59 and HHUA cells were treated with 3 μM CDDP and 200 μM SAS and 0.5 μM CDDP and 300 μM SAS, respectively. Values shown represent means + SD. ^*^ *P* < 0.05.

GSH is synthesized from Cys, Glu, and Gly in a series of metabolic steps. To assess the differences in the effect of SAS on GSH depletion between USC and EmC cells, we measured the expression of proteins associated with GSH synthesis. HepG2 cells, which express these proteins, were used as positive controls. Immunoblot analysis showed that xCT expression was increased in the USPC-1, SPAC, and HEC1A cell lines (Figure 4A). GCLC expression was slightly decreased in the HHUA and HEC59 cell lines (Figure 4B). Finally, we measured the expression of CGL and synthesized Cys from Met via the trans-sulfuration pathway. CGL expression levels were increased in the HEC59, HHUA, and HEC1A cell lines (Figure 4C). Upregulation of the trans-sulfuration pathway rescues cells from cell death induced by xCT inhibition [25]. Therefore, we investigated the effect of SAS on CGL expression in the USPC-1 and HHUA cell lines. SAS increased CGL expression in both cell lines (Figure 4D). Next, we evaluated the effect of the CGL inhibitor, PPG. PPG enhanced SAS- or combined CDDP plus SAS-mediated cell growth inhibition compared with either treatment without PPG in USPC-1 cells (Figure 4E, upper left panels). In HEC59 cells, PPG significantly increased growth inhibition induced by treatment with CDDP plus SAS (Figure 4E, upper right panels). In HHUA cells, PPG treatment significantly inhibited cell proliferation induced by treatment with CDDP or SAS alone or with CDDP plus SAS (Figure 4E, under left panels). Collectively, the inhibition of xCT activated the trans-sulfuration pathway in USC and EmC cells. However, SAS exhibited GSH depletion and enhanced CDDP-mediated cytotoxicity because of elevated levels of xCT expression and decreased levels of CGL expression in USC cells. One reason why SAS showed no GSH depletion in EmC cells may involve low xCT expression and activation of the trans-sulfuration pathway, which is composed of CGL. These results suggest that GSH synthesis primarily depends on the trans-sulfuration pathway in EmC cells. Taken together, the expression of xCT and CGL may be a predictive marker for the effect of SAS.

### Effect of CDDP plus SAS on the growth of tumor xenografts

**Figure 5:**
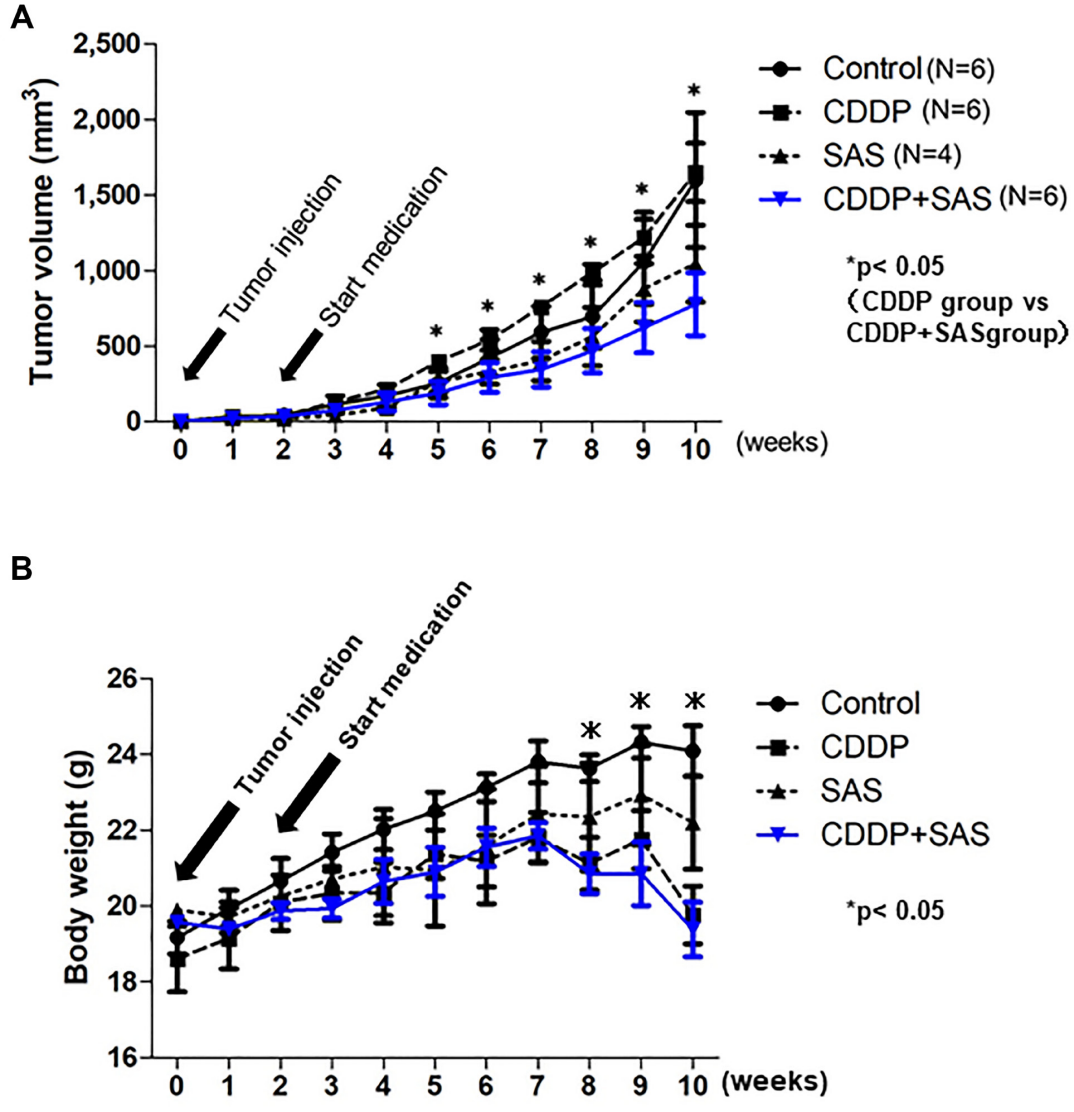
Cisplatin (CDDP) and Sulfasalazine (SAS) suppress xenograft tumor growth. (**A**) Time course of the volume of tumors formed by USPC-1 cells in hairless SCID mice treated with CDDP (3 mg/kg) and/or SAS (250 mg/kg). Values shown represent means ± SD. (**B**) Time course of the weight of mice. Values shown represent means ± SD. ^*^ *P* < 0.05, compared with control group mice.

To confirm whether SAS enhances the efficacy of CDDP in USC xenograft models, we administered CDDP and/or SAS to hairless SCID mice subcutaneously inoculated with USPC-1 cells (5 × 10^6^). We divided 24 hairless SCID mice into four groups. We excluded two mice because one mouse belonging to only the SAS treatment group exhibited no tumor formation one week after injection, whereas the other belonging to only the SAS treatment group died by subcutaneous emphysema resulting from a technical problem. Five weeks after injection, tumor growth in mice treated with CDDP plus SAS was suppressed compared with that in mice treated with DMSO, CDDP, and SAS. A one-way ANOVA with the Bonferroni post-hoc test indicated that the tumor volume tended to remain smaller compared with the other groups for the next 5 weeks (*p* = 0.37) (Figure 5A). The *t*-test indicated that tumor growth was significantly suppressed compared with that observed in mice treated with CDDP alone 3 weeks after beginning treatment with CDDP plus SAS. With respect to side effects, body weight in the SAS plus CDDP treatment group was significantly lower (19.5%) compared with that in the control group (*p* < 0.05). However, there was no difference in body weight between the CDDP group and CDDP plus SAS group (Figure 5B). No other side effects, such as eruption and diarrhea, were observed.

## DISCUSSION

In the current study, we investigated whether targeting the Glu-cystine transporter, xCT, is a promising strategy to overcome endometrial cancer. The xCT inhibitor, SAS, decreased GSH and induced apoptosis by co-treatment with CDDP in USC. The route of accumulating Cys, a component of GSH, determined the effectiveness of SAS (Supplementary Figure 1). Our study is the first to demonstrate a relationship between the GSH synthesis pathway and the efficacy of SAS in endometrial cancer.

Compared with the GSH concentration of endometrial cancer cell lines, the GSH concentration in USC cell lines was higher compared with that in EmC cell lines. We also showed that the effect of SAS on cytotoxicity was more significant in USC cells compared with EmC cells, and SAS enhanced CDDP cytotoxicity in USC cell lines and in a xenograft model, although the effect of SAS on cytotoxicity was not observed in EmC cells. Interestingly, the two types of endometrial cancer showed a different responsiveness to SAS. Ninety percent of type II endometrial cancer cases represented by serous carcinoma have p53 mutations versus 10–20% of type I endometrial cancers represented by EmC [26]. The p53 protein is an important tumor suppressor that mediates transcriptional repression of SLC7A11, a component of xCT [27]. By repressing SLC7A11 transcription, p53 reduces cystine uptake, which in turn, limits the production of intracellular GSH [27]. The high rate of p53 mutation in USC may affect intracellular GSH levels and the effects of SAS.

In general, anticancer agents, such as CDDP produce ROS in cancer cells and induce apoptosis [14]. However, GSH scavenges free radicals and other ROS [15, 28]. We predicted that SAS enhances the efficacy of CDDP, which accumulates ROS and induces apoptosis. We observed that the cell death ratio and ROS levels were increased in the CDDP plus SAS group; therefore, the combination of CDDP plus SAS resulted in ROS accumulation and induced cell death in USPC-1 cells. The sensitization effect of SAS on CDDP induced apoptosis, which was accompanied by enhanced PARP cleavage, was blocked by the pan-caspase inhibitor, Z-VAD-FMK. SAS has been widely reported as an inhibitor of xCT; however, the effects on GSH levels in cancer cells treated with SAS were different for each cell line [29–31]. Given our findings that the administration of SAS reduces GSH levels in USPC-1, but does not affect GSH levels in HHUA, we hypothesized that GSH depletion contributes to cell death by CDDP. As an agent used to deplete GSH, we focused on BSO, which inhibits GCLC, a key enzyme in GSH production. GCLC is a rate-limiting enzyme that generates γ-Glu-Cys from Glu and Cys to produce GSH [32]. BSO, a GCLC inhibitor, is used as a drug to deplete GSH, but this drug results in GSH reduction, not only in cancers, but also in normal tissues [33]. Moreover, a phase I study did not achieve an adequate reduction of GSH levels [34] and no phase II clinical trials have been conducted to date. Our study revealed that BSO reduced GSH levels not only in USPC-1 cells, but also in EmC cells, and induced apoptosis in combination with CDDP. Cell death is not induced in combination with CDDP under conditions in which GSH is not reduced. This suggests that GSH depletion induces ROS-related apoptosis in endometrial cancer cells in combination with CDDP.

To assess differences in the efficacy of SAS, we focused on the expression of proteins related to GSH synthesis. In a previous report, most of the Cys is transported into cancer cells via xCT, but a significant percentage may be synthesized from Met, via the trans-sulfuration pathway in glioma cell lines. The xCT inhibitor depleted GSH to 51% of the control, whereas inhibition of the trans-sulfuration pathway depleted GSH to 77% of the control [35]. In another report, the trans-sulfuration pathway compensated Cys depletion when the uptake through xCT was inhibited [36], and upregulation of the trans-sulfuration pathway rendered cells insensitive to death induced by xCT inhibition [25]. In the present study, we demonstrated that xCT expression was high and CGL expression was low in USC cell lines. However, in EmC cell lines, xCT expression was low or the expression of both CGL and xCT was high. We showed that CGL expression was increased by SAS treatment in USPC-1 and HHUA cells; therefore, the trans-sulfuration pathway may compensate for xCT inhibition. Despite the compensation of the trans-sulfuration pathway, USC cells with high xCT and low CGL expression underwent apoptosis by SAS and CDDP treatment. In contrast, because of the influence of the compensation of trans-sulfuration pathway, SAS requires trans-sulfuration pathway inhibition with PPG for cell growth suppression in EmC cells with low xCT or high CGL expression. These results suggest that USC cells rely on xCT to obtain Cys, in contrast to EmC cells in the trans-sulfuration pathway. Therefore, the combination of SAS and CDDP had an impact on USC cell viability and the expression of xCT and CGL can predict the efficacy of SAS.

GSH is a well-known target for overcoming treatment-resistant cancers. GSH level is dependent on the turnover of NADPH, which reduces glutathione disulfide (GSSG) to GSH. The xCT transporter is important for GSH synthesis and may be targeted for treatment. The xCT inhibitor, SAS, has long been used in clinical practice for the treatment of rheumatoid arthritis and inflammatory bowel disease, and it has also been used to treat various cancers. To date, several clinical trials have been carried out; however, the effect of SAS remains controversial. A phase I/II study for recurrent or progressive malignant glioma was terminated because of a lack of clinical response and a high frequency of adverse effects, including increased neurologic deficit, myelosuppression, and proteinuria [37]. However, in advanced non-small-cell lung cancer patients, the combination of SAS exhibited a much higher overall response rate and longer median progression-free survival than previously reported with CDDP or pemetrexed alone [38]. Previous clinical trials did not stratify patients by predicting SAS efficacy based on the expression of the GSH synthesis pathway, such as xCT and CGL expression, which may have resulted in a lack of efficacy. Additionally, NADPH has been suggested to be involved in GSH reduction; thus, the NADPH metabolic pathway could be a potential biomarker for GSH targeting therapy.

Our results indicate that the expression of proteins related to GSH synthesis, especially xCT and CGL, varied by histopathology in endometrial cancer and that the efficacy of SAS in enhancing CDDP cytotoxicity depends on these proteins. The trans-sulfuration pathway may have affected the therapeutic effect of SAS in clinical studies. An approach based on GSH synthesis status may be used to stratify patients to improve the therapeutic efficacy of SAS. The expression of xCT and CGL can be used as predictive markers for the efficacy of SAS and clinical biomarkers for individualized therapy.

## MATERIALS AND METHODS

### Antibodies and reagents

CDDP, SAS, and DL-propargylglycine (PPG) were purchased from Sigma-Aldrich (St. Louis, MO, USA). Buthionine sulfoximine (BSO) was purchased from Cayman Chemical (Ann Arbor, MI, USA). Z-Val-Ala-Asp(OMe)-CH2F (Z-VAD-FMK) was purchased from Peptide Institute, Inc. (Osaka, Japan). Anti-xCT antibody (ab37185) was purchased from Abcam (Cambridge, UK). Anti-cleaved poly (ADP-ribosyl) polymerase (PARP) antibody (#9541) was purchased from Cell Signaling Technology, Inc. (Beverly, MA, USA). Anti-GCLC antibody (GTX16315) was purchased from Gene Tex, Inc. (San Antonio, TX, USA). Anti-cystathionine gamma lyase (CGL) antibody (12217-1-AP) was purchased from Proteintech (San Antonio, TX, USA).

### Cell culture

The human USC cell lines, USPC-1 and SPAC, were kindly provided by Dr. Yaegashi, Department of Gynecology and Obstetrics, Tohoku University (Miyagi, Japan). USPC-1 was maintained in RPMI 1640 medium (Thermo Fisher Scientific, Inc.) supplemented with 10% fetal bovine serum (FBS; Cytiva), GlutaMAXTM I (2 mM L-glutamine; Thermo Fisher Scientific, Inc.) and an antibiotic/antimycotic mixture (100 U/ml penicillin, 100 μg/ml streptomycin, and 250 ng/ml amphotericin B; Thermo Fisher Scientific, Inc.) at 37°C in a humidified atmosphere with 5% CO_2_. SPAC was maintained in RPMI 1640 medium supplemented with 10% FBS and 1% penicillin/streptomycin (Sigma-Aldrich; Merck) at 37°C in a humidified atmosphere with 5% CO_2_. The human EmC cell lines, HEC59 and HEC265, have been registered at the JCRB cell bank as JCRB1120 and JCRB1142, respectively. The human EmC cell line, HEC1A, was kindly provided by Dr. Murata, Department of Gynecology and Obstetrics, Osaka University (Osaka, Japan). HEC59, HEC265, and HEC1A were maintained in Eagle’s Minimum Essential Medium (Wako Pure Chemical Industries, Ltd., Osaka, Japan) containing 15% FBS at 37°C in a humidified atmosphere with 5% CO_2_. HHUA, a human EmC cell line, has been registered at the RIKEN Cell Bank as RCB0658 and maintained in Ham’s F12 medium (Wako Pure Chemical Industries, Ltd., Osaka, Japan) containing 15% FBS at 37°C in a humidified atmosphere with 5% CO_2_.

### GSH analysis

Intracellular GSH levels were measured using the GSH-Glo™ luminescent-based assay (Promega Corporation). Cells were seeded into white 96-well plates at 5 × 10^3^ cells per well and incubated at 37°C for 2.5 h. Luminescent signal was measured using Thermo Scientific Varioskan^®^ Flash (Thermo Fisher Scientific K.K., Tokyo, Japan).

### Cell proliferation assay

Cells were seeded into 96-well plates at 1 × 10^3^ cells per well and incubated at 37°C for 24 h, then treated with anticancer agents and incubated at 37°C for 48 h. The Cell Titer 96^®^ AQ_ueous_ One Solution Cell Proliferation Assay (MTS assay; Promega Corporation) was used to measure cell viability. We determined the drug concentrations based on the half-maximal inhibitory concentration (IC_50_) values. Each dose was lower than the IC_50_ value. The IC_50_ values were calculated using the following formula (13): IC_50_ = 10^[log(A/B) × (50-C)]/[(D-C) + log(B)]^, where A and B are the corresponding concentrations of the tested drug directly above and below 50% inhibition, respectively, and C and D correspond to the percentage of inhibition directly below and above 50% inhibition, respectively.

### Cell death assay

Cell death was assessed by Hoechst 33342 (Invitrogen; Thermo Fisher Scientific, Inc.) and propidium iodide (PI; Invitrogen; Thermo Fisher Scientific, Inc.) co-staining. Cells were seeded into 12-well plates at 2 × 10^4^ cells per well and incubated at 37°C for 24 h. After treatment, the cells were stained with 5 μg/mL Hoechst 33342 and 0.5 μg/mL PI for 15 min, then visualized and scored using a fluorescence microscope (Leica DMI 3000B). The percentage of cell death was calculated as the ratio of PI-positive cells in relation to the total cells stained with Hoechst 33342. This calculation was made from 3 random fields per well.

### Detection and measurement of intracellular ROS

Cells were washed twice with PBS and resuspended in PBS. Cells were treated with 10 μM 2′,7′-dichlorofluorescein diacetate (DCF-DA; Sigma-Aldrich; Merck) for 10 min. The probes were shielded from light during DCF-DA treatment. Cells exhibiting a signal for DCF above the gate established by the isotype control were deemed ROS-positive. The cells were then subjected to flow cytometric analysis for quantification of the intensity of DCF fluorescence using a FACSCantTM II Flow Cytometer (BD Biosciences, Franklin Lakes, NJ, USA). The resulting data were analyzed using FlowJo software, version 7.6.5 (Treestar Inc., Ashland, OR, USA).

### Immunoblot analysis

Cells were washed with ice-cold PBS and lysed in RIPA buffer (FUJIFILM Wako Pure Chemical Corporation, Osaka, Japan). After centrifugation for 10 min at 14,000 × g at 4°C, the supernatants were recovered as the cell lysates, and protein concentration was measured using the DC™ protein assay kit (Bio-Rad Laboratories, Inc., Hercules, CA, USA). Cell lysates containing equal amounts of protein were separated by sodium dodecyl sulfate-polyacrylamide gel electrophoresis and transferred to a polyvinylidene difluoride membrane. The membrane was probed with primary antibody followed by the corresponding horseradish peroxidase-conjugated secondary antibody according to the protocol recommended by the manufacturer of each antibody. Immunoreactivity bands were visualized using the ECL Prime Western Blotting Detection Reagent (GE Healthcare Life Sciences, Buckinghamshire, England).

### *In vivo* studies

A subcutaneous xenograft model was established by suspending USPC-1 cells (5 × 10^6^ viable cells) in 200 μL of PBS after, determining cell viability, and injecting them into the subcutaneous tissue of 6-week-old female Crlj: SHO-Prkd^scid^Hr^hr^ Hairless SCID mice (Charles River Laboratories JAPAN, Inc., Kanagawa, Japan). After implantation, the mice were monitored for general health status and the presence tumors. Tumor volume was determined by measuring tumor diameters (the measurement of 2 perpendicular axes of tumors) and calculated as 1/2 × (larger diameter) × (smaller diameter)^2^. We determined the correct dose of CDDP and SAS according to a previous report to maximize their antitumor effects and minimize adverse effects [21]. Mice were treated by intraperitoneal injection of CDDP (3 mg/kg) or DMSO weekly and administered an SAS suspension (250 mg/kg) orally five times per week. Tumor-bearing mice (*n* = 24) were randomly assigned into four groups as follows: group 1, administered DMSO weekly; group 2, CDDP weekly: group 3, SAS 5 times per week; group 4, CDDP weekly and SAS 5 times per week for 8 weeks. Treatment was initiated 2 weeks after cancer cell injection. The mice were sacrificed at week 9 after the start of treatment. The animal experiments conducted in this study were performed under the protocol approved by the Animal Research Committee of Yamagata University (No. 31009).

### Statistical analysis

The results are expressed as the means and standard deviation (SD), and differences were compared using a 2-tailed Student’s *t*-test or one-way ANOVA with Bonferroni’s post-hoc test. *P*-values less than 0.05 were considered statistically significant and indicated with asterisks in the figures.

## SUPPLEMENTARY MATERIALS


