# Supplementary material for: Impact of the glutathione synthesis pathway on sulfasalazine-treated endometrial cancer
Source: Oncotarget. 2022 Jan 26;13:224–36. doi: 10.18632/oncotarget.28185 (PMC8794577; doi:10.18632/oncotarget.28185)
Supplement: Supplementary file 1 [file oncotarget-13-28185-s001.pdf]

Impact of the glutathione synthesis pathway on sulfasalazine-treated endometrial cancer

SUPPLEMENTARY MATERIALS

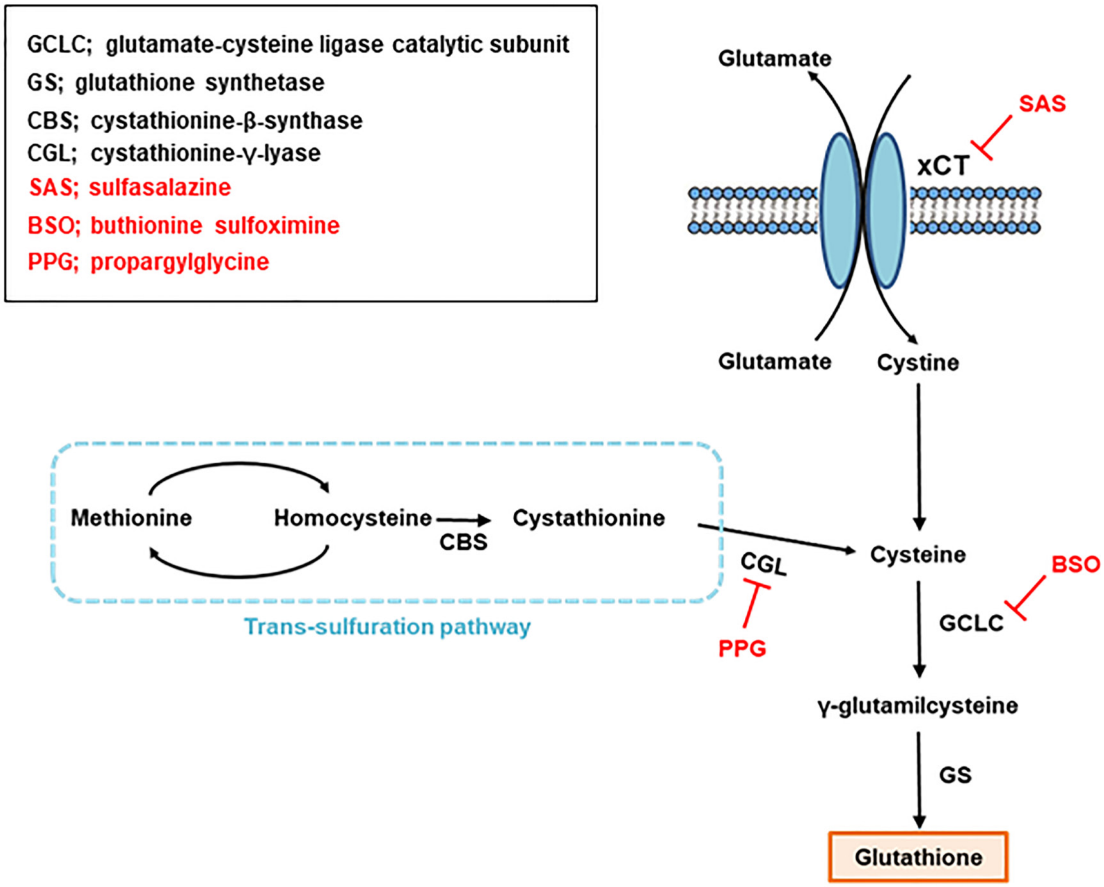

Supplementary Figure 1: Glutathione (GSH) synthesis pathway. GSH is a tripeptide composed of Glu, Cys, and Gly. Cys is transported into cancer cells via the Glu-Cystine transporter, xCT, or derived from Met via the trans-sulfuration pathway. GSH synthesis is regulated by GCLC activity, Cys availability, and GSH feedback inhibition.
